# Motor Demands Constrain Cognitive Rule Structures

**DOI:** 10.1371/journal.pcbi.1004785

**Published:** 2016-03-11

**Authors:** Anne Gabrielle Eva Collins, Michael Joshua Frank

**Affiliations:** 1 Department of Cognitive, Linguistic and Psychological Sciences, Brown Institute for Brain Science, Brown University, Providence, Rhode Island, United States of America; 2 Department of Psychology, University of California, Berkeley, Berkeley, California, United States of America; Brain and Spine Institute (ICM), FRANCE

## Abstract

Study of human executive function focuses on our ability to represent cognitive rules independently of stimulus or response modality. However, recent findings suggest that executive functions cannot be modularized separately from perceptual and motor systems, and that they instead scaffold on top of motor action selection. Here we investigate whether patterns of motor demands influence how participants choose to implement abstract rule structures. In a learning task that requires integrating two stimulus dimensions for determining appropriate responses, subjects typically structure the problem hierarchically, using one dimension to cue the task-set and the other to cue the response given the task-set. However, the choice of which dimension to use at each level can be arbitrary. We hypothesized that the specific structure subjects adopt would be constrained by the motor patterns afforded within each rule. Across four independent data-sets, we show that subjects create rule structures that afford motor clustering, preferring structures in which adjacent motor actions are valid within each task-set. In a fifth data-set using instructed rules, this bias was strong enough to counteract the well-known task switch-cost when instructions were incongruent with motor clustering. Computational simulations confirm that observed biases can be explained by leveraging overlap in cortical motor representations to improve outcome prediction and hence infer the structure to be learned. These results highlight the importance of sensorimotor constraints in abstract rule formation and shed light on why humans have strong biases to invent structure even when it does not exist.

## Author Summary

Humans’ ability to create abstract rule structures contributes greatly to intelligence and higher cognitive functions as it affords flexible re-use across various sensory-motor transformations. However, how such rule structures develop through learning is poorly understood. Models of this process imply that cognitive rule learning scaffolds on top of mechanisms that support motor action selection learning. Here, we show that the form of motor demands across multiple stimulus-action-outcome influences the form of the abstract rule structures that are created. Our research highlights the strong reciprocal influences of low and high-level cognition, offers further insight into how we learn hierarchical structures, and reminds us that how we act constrains how we think.

## Introduction

Making decisions in the complex environment of daily life often requires cognitive control to flexibly adjust our behavior: the appropriate reaction to different sensory events often depends on the current context, our goals, etc. For example, while driving, if you see a red light on your street, you will stop; if it turns green, you will go–but the opposite actions apply when the red/green light is in the intersecting street. The cognitive control literature has shown that humans use contextual cues to select abstract sets of rules (or task-sets) that are behaviorally relevant [1,2]. Specifically, cognitive control relies on a cascading hierarchical structure, where at a more abstract level, subjects choose task-sets appropriate to the *context*, which then constrain our action choices in response to lower-level, less abstract *stimuli* [3,4]. Thus, we use the term “context” to refer to those features that cue abstract task-set, and the term “stimulus” to refer to features that cue the appropriate response conditioned on the selected task-set.

In the study of executive functions, researchers tend to focus on discretized aspects of decision making–often assuming that perception systems have transformed complex, multi-dimensional sensory signals into reduced, discrete stimuli (e.g., a red circle), and that given this percept, the executive system selects among a few discrete options (e.g., left vs. right), which are then implemented by the motor system. However, the field of embodied or grounded cognition [5–7] offers strong hints that this model is over-simplified, emphasizing instead that executive functions evolved for the control of action in continuous time [5], and are thus scaffolded on existing sensorimotor processing systems. One hint that executive functions are grounded in sensorimotor processing [8,9], is the inability to isolate functionally distinct neural systems for executive functions from motor execution, such that for example, cerebellum, mostly thought of as a motor system, is strongly involved in cognitive control [10,11]. Another hint comes from the organization of cortico-basal ganglia loops, where prefrontal-striatal connectivity parallels that of premotor-cortex for action selection, with hierarchical influence of more anterior loops representing cognitive rules over more posterior loops involved in selecting motor actions [12–15]. Through learning, the consequences of motor actions are leveraged not only to improve future motor choices, but also to improve selection of higher order rules [12–16] again implying a scaffolding of more abstract “cognitive” action selection onto motor action selection. Similarly, striatal dopamine manipulations have analogous effects on reinforcement learning and cognitive action selection [17,18]. However, to date, we know of no direct evidence to indicate that constraints within the motor system influence the way our brain represents the more abstract variables needed for cognitive control, such as task-sets. Here, we propose that an intrinsic constraint of motor action representations strongly influences how we create representations of abstract hierarchical rule structures, both during learning and while applying instructed rules.

Recent research has shown that humans can acquire abstract rule representations through reinforcement learning, using only reward feedback. Structured rules are discovered when they are present in the task and helpful for learning and generalization [19–21], and there is evidence that subjects look for and create such structure, even when it is not beneficial [15,16,22]. However, creating structure is potentially effortful and costly: a learner needs not only to discover the appropriate set of rules given a defined set of “higher-level” *contexts* that cue these rules and “lower-level” *stimuli* that cue motor actions, but also how to organize the complex multidimensional world into structured components (contexts, stimuli) in the first place. Indeed, more than one hierarchical structure representation of the environment is possible and it is not always evident which features should be contexts cuing task-sets and which should be considered lower level stimuli. Often, one hierarchical structure is more useful than others because it can afford greater generalization to novel contexts (e.g., if multiple contexts cue the same task-set, they can be clustered together [15], Collins & Frank, submitted). For example, the rule to apply for red and green traffic lights is the same whether the intersecting street comes from the left or the right, such that using the position for context allows generalization, and thus simplifies the problem.

Here, we investigated whether motor patterns provide an additional constraint on imposing hierarchical cognitive rule structures. To this effect, we take advantage of motor representational structure in motor cortex. Finger movement representations in M1, while globally somatotopic, are widely overlapping for neighboring fingers [23,24], reflecting the natural statistics of hand movement structure [24]. This representational constraint may lead to pre-activation of neural networks representing frequently co-activated fingers (motor synergies [25,26]), thus facilitating further choices with those fingers. For example, cues that allow for motor preparation of adjacent, rather than segregated, finger presses facilitate action [27–29].

Therefore, we investigated whether this motor constraint could influence the nature of hierarchical rule structures when actions are selected via finger presses on a computer keyboard. Specifically, we hypothesized that subjects might naturally cluster together sets of stimulus-action associations that were more similar in motor space, for example, that involved adjacent fingers, within each task-set. This would then lead to a natural constraint on the creation of abstract rule structure: subjects should treat those feature dimensions that afford motor clustering as lower order stimulus (allowing clustering within rules), and those that afford less motor clustering as higher order context.

To test our hypothesis, we tested subjects in a structure-learning task, in which we previously showed that subjects create one of two possible hierarchical structures, even when they could learn the task as well without imposing structure at all. We show in two new data sets that the potential for motor clustering within a task-set influences the nature of the structure created. We further confirm and replicate this finding in a novel re-analysis of two published data-sets [15,22]. We then use computational modeling to investigate how these biases may emerge. Simulations show that assuming that low level motor representational biases simply influence action selection is not sufficient to account for structure learning biases observed. Rather, we implement a simple computational mechanism based on the motor scaffolding hypothesis that assumes that motor representational constraints affect outcome predictions, and thus inference of structure. We show that this mechanism can account for subject’s biases in structure learning. Finally, we show that this bias may be strong enough to induce subjects to restructure rules, even if they do not need to learn them, but know them through practice and instructions, when such rules are set up such that they conflict with motor clustering constraints.

## Results

**Fig 1:**
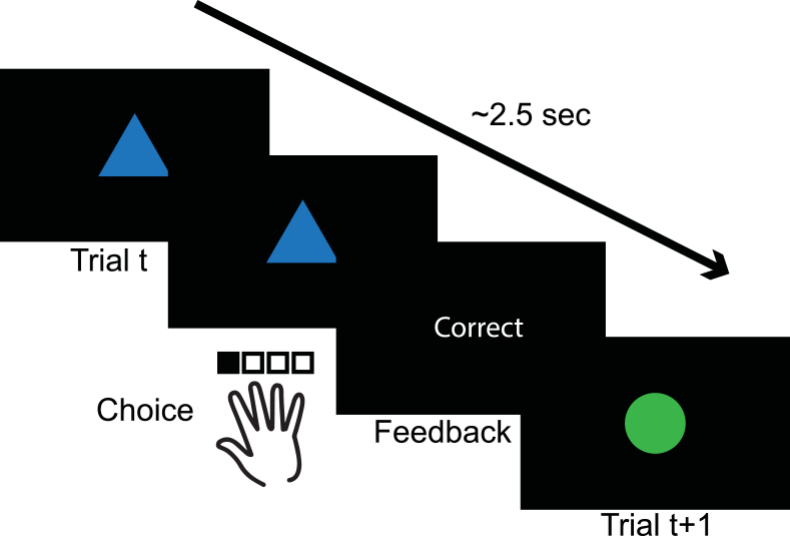
Experimental design. In all tasks subjects saw a two-feature visual input pattern (e.g. a colored shape), pressed one of four keys, and were provided feedback, before going on to a randomly chosen next stimulus. In experiments 1–4, subjects used the truthful feedback to learn which actions were correct for each input. In the last experiment, subjects were instructed the correct associations and practiced them (Fig 6), but still received feedback.

**Fig 2:**
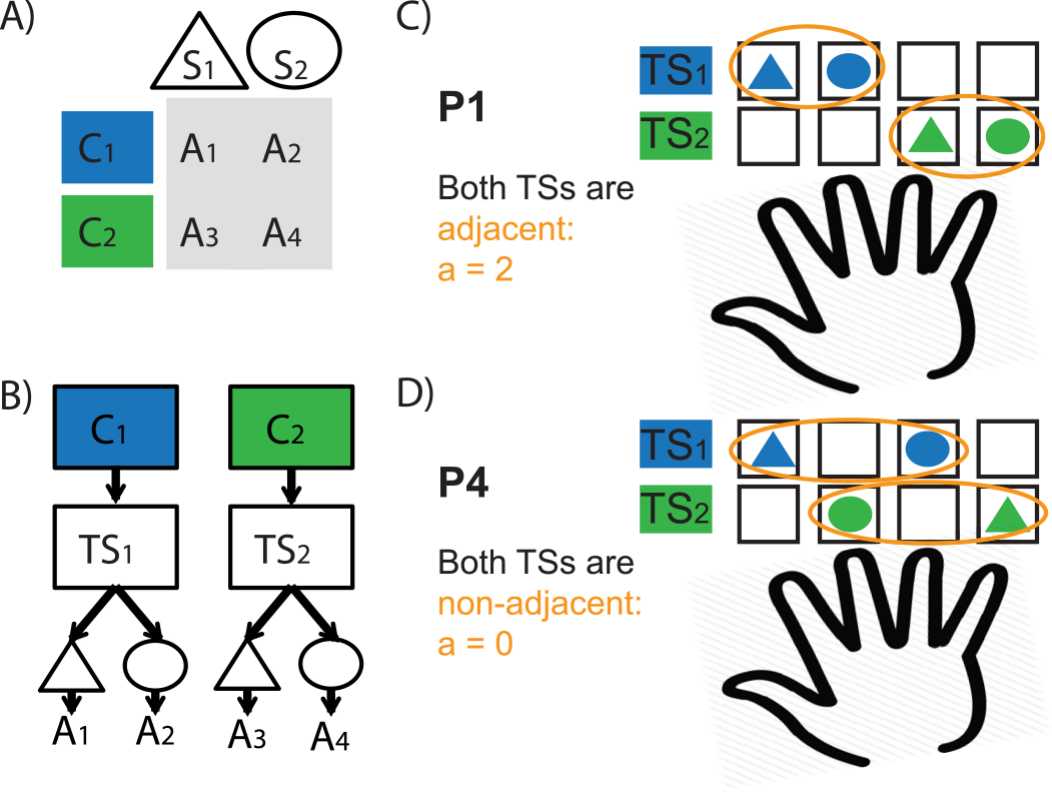
Motor patterns and hierarchical structure. **A.** Correct action contingencies: visual input patterns are two-dimensional images, e.g. colored-shapes. **B** this can be represented hierarchically, with one of the features (here, e.g. color) serving as a context cueing a task-set (TS), and the other as a stimulus for which an action is selected given the TS. **C,D.** Depending on mapping of actions A1-A4 to individual fingers, different learning problems afford different motor patterns (in this case we show two problems both assuming the same hierarchical structure). **C.** In this configuration, motor patterns within both task-sets are “adjacent” (the correct key presses for stimuli are on adjacent fingers), leading to an *adjacency bonus* a = 1+1. **D.** Here, motor patterns required for both task-sets are *non-adjacent* (a = 0).

In a first experiment, 22 subjects performed 6 independent blocks of a reinforcement learning task shown previously to induce creation of hierarchical rule structures [15,22]. Specifically, they used truthful correct/incorrect feedback to learn to select the correct one out of four possible actions (button presses with four fingers of the dominant hand) for each of four distinct visual input patterns (colored shapes, see Figs 1A and 2), presented in pseudo-randomized order.

**Fig 3:**
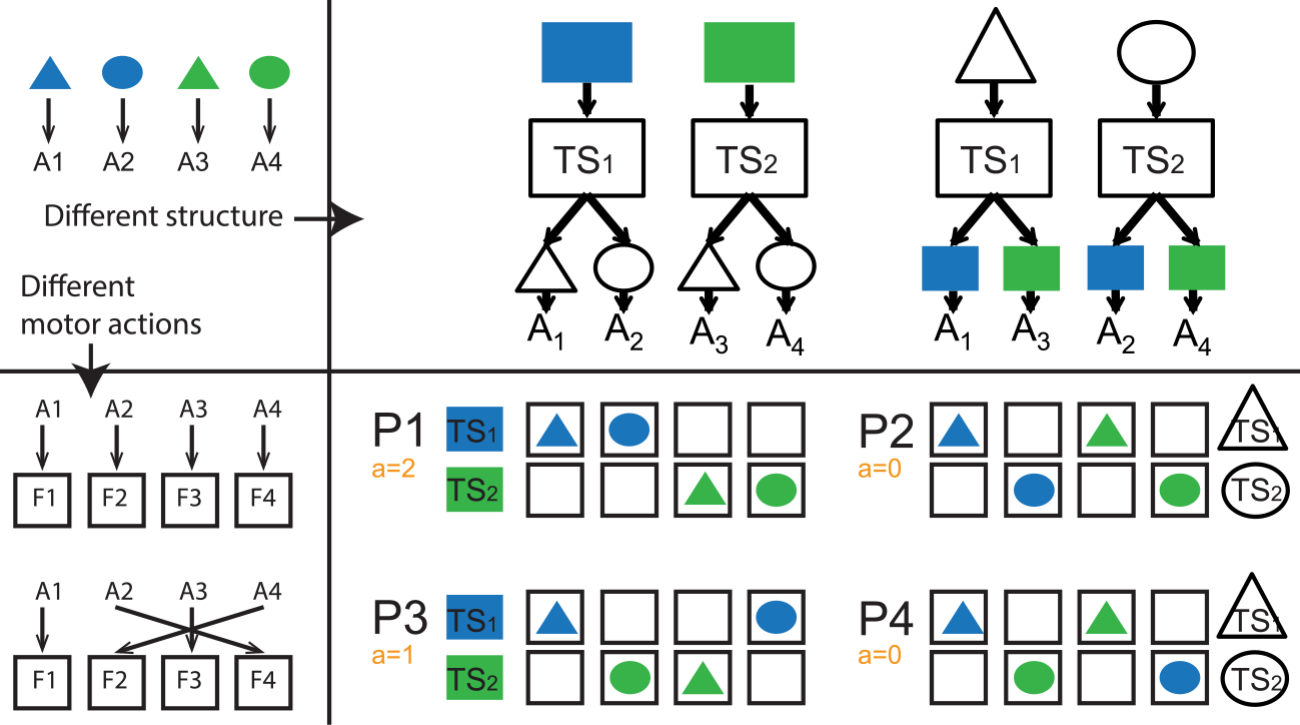
Hierarchical structure representation affects motor patterns. For a single abstract learning problem (top left), different assignment of actions A1-A4 to actual finger presses F1-F4 (left), leads to different patterns of motor assignment across task-sets. A given assignment will lead to one of two possible configurations, depending on which structure is chosen (color on top or shape on top). Bottom right Each motor configuration (P1-P4) is depicted assuming subjects structure the task with task-sets selected according to color (P1-P3 on left) or shape (P2-P4 on right), with the context that cues each task-set shown (e.g.–blue for TS1 of P1, triangle for TS1 of P4). For each pattern, the adjacency bonus *a* (indicating whether a task-set’s actions are adjacent) and is indicated. Note in particular that neighboring configurations (P1 and P2, P3 and P4) correspond to the same colored-shape action contingencies, grouped into different hierarchical structure representations, and that P1 and P3 have a higher bonus than their counterpart. Role of color and shape is counterbalanced to ensure that the color-as context structure does not always correspond to clustering configurations P1/P3, as is shown here. Note that there is one other possible set of configurations (P5/6) not displayed here, because other factors contribute to an independent bias in these cases. See S3 Fig for results including this configuration.

Although such problems could be learned by standard learning algorithms assuming that each input pattern is a distinct state that simply combines color and shape, we showed in previous research [15,22,30] that subjects impose structure onto such learning problems using hierarchical rules, such that one dimension (e.g., color in Fig 2B) serves as a context that cues a task-set, and the other (e.g., shape in Fig 2B) as a stimulus that directs action selection under the constraint of the current task-set. Imposing such structure facilitates transfer of learned task-sets to novel contexts [15,22,30] and speeds learning when multiple contexts cue the same task-set. Fig 3 shows that two structures are possible for a single learning problem (e.g. with colors as the contexts cueing task-sets, or shapes as the contexts cueing task-sets). Depending on the specific mapping of actions onto four fingers of the right hand, a given structure may elicit more or less efficient motor clustering patterns; two contrasting examples are given in Fig 2, each when color is used as context.

We define a measure based on the motor representation biases introduced above: the *adjacency bonus* indicated whether a task-set cue clustered motor action (if actions for both stimuli require key presses from adjacent fingers within each task-set; see Fig 2 and Methods).

For a fixed assignment of actions to fingers, contrasting the two potential hierarchical structures (color vs. shape structure) leads to different motor patterns: P1 vs. P2, or P3 vs. P4 (Fig 3). We test here the theory that subjects tend to create the structure associated with greatest affordance for motor clustering, and specifically hypothesized that subjects would select structures P1 over P2 (a = 2 vs. 0), and P3 over P4 (a = 1 vs. 0).

To identify which structure was created, we relied on reaction-time task-switch costs, one of the most reliable findings in cognitive psychology [1]. Due to the additional cognitive demands associated with updating task-sets (thought to relate to processing within prefrontal cortex), response times are slower when the task-set changes from the previous trial, compared to when it repeats, and this slowing is greater than the corresponding cost associated with changes in stimuli within a task-set [1,15]; reaction time switch-cost measures this relative slowing. This measure allows us to differentiate the structures: those subjects adopting color-on-top structures should exhibit greater RT switch costs for switches in color than shape, and vice versa. We thus use the difference between reaction time switch-cost of each dimension as evidence in favor of either structure. We have previously validated this measure in that it was independently predictive of subjects’ ability to transfer the inferred structure to novel contexts that involve the same structure, and was also predictive of neural signals reflecting the same hierarchical structure [15,22].

To evaluate whether the selected structure was affected by potential for motor clustering, we define the *motor clustering switch-cost* as the difference between the switch costs defined by the structure affording greater motor clustering and that affording less clustering, e.g., switch cost for P1 or P3 minus the switch-cost for P2 or P4. Thus, our prediction that subjects would be more likely to create the hierarchical rule structure that affords motor clustering translated into a prediction that the motor clustering switch-cost should be positive.

**Fig 4:**
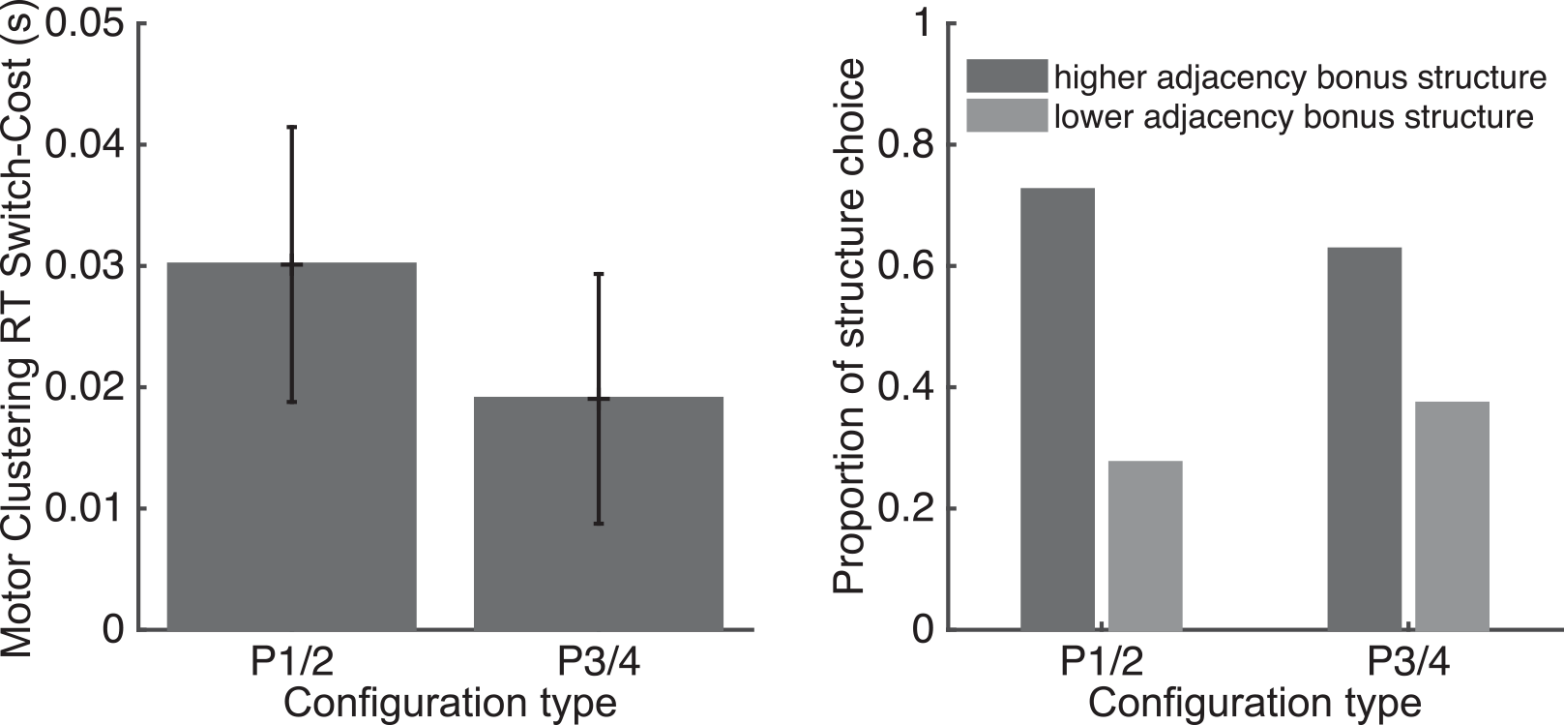
Subjects favor creation of structure corresponding to motor clustering configuration. **Left.** Motor clustering bonus, in terms of switch-costs differences, for each configuration type (error bars correspond to standard error). **Right.** Proportion of learning blocks in which subjects were identified as having represented the task with the higher adjacency bonus structure (dark bars) or the lower one (light).

Results confirmed our predictions: we found that Motor clustering RT switch cost was significantly greater than 0 (Fig 4 left, p = 0.0007, t(21) = 4.14). This measure did not differ between configuration types P1/2 and P3/4 (t = 0.36, ns). Furthermore, individual learning problems were significantly more likely to be identified as subjects having created the structure affording more motor clustering (P1-3) than the one affording less (P2-4, binomial test p = 0.0012; 66 out of 99 problems; Fig 4 right).

We sought to replicate this result in three further data-sets, using nearly identical experimental designs (two of which were previously published but where motor patterns were randomized and not assessed [15,22]), with only one learning block per subject. Identical analysis performed over all three datasets confirmed the previous results: reaction time-switch cost was significantly higher for the structure with higher adjacency bonus, measured by a positive motor-clustering switch-cost (t(91) = 3.96; p = 0.00015; S4 Fig). There was no effect of experiment on motor-clustering switch-cost (F = 0.34, p = 0.71), nor was there an effect of configuration type (P1/3 vs. P2/4; p = 0.13; S4 Fig). Furthermore, 61 (vs. 31) out of 92 subjects had a positive motor-clustering switch-cost, labeling them as having created the higher adjacency bonus structure (S4 Fig, right). This is significantly more than expected by a balanced random distribution (p = 0.0023, binomial test), supporting our previous finding.

We thus observed across four independent data sets that subjects were more likely to create hierarchical task structure that lead to grouped task sets, highlighting an influence of motor choices on abstract representations known to be crucial for executive functions [1,31]. Note that this bias has no bearing on the subjects’ actual motor choices: independently of how they implement the task, the sequence of valid motor actions to take is the same. Thus, we believe that this phenomenon emerges from strong biases highlighting constraints in how our brain represents rules. We next investigate the mechanisms that could lead to those biases.

We previously proposed a computational model of hierarchical structure learning [15]. The model simulates creation of task-set structure, allowing for clustering of multiple contexts that link to task-sets and which guide lower order stimulus-action-outcome associations. Moreover, it can infer the type of structure, e.g. which features are indicative of contexts that cue latent task sets and which should be considered low order stimuli. It does so by considering which structure is best able to predict the observed outcomes (here, reinforcement feedback) and assumes that structure describes the current environment most efficiently, and allowing for transfer of structures to novel contexts.

**Fig 5:**
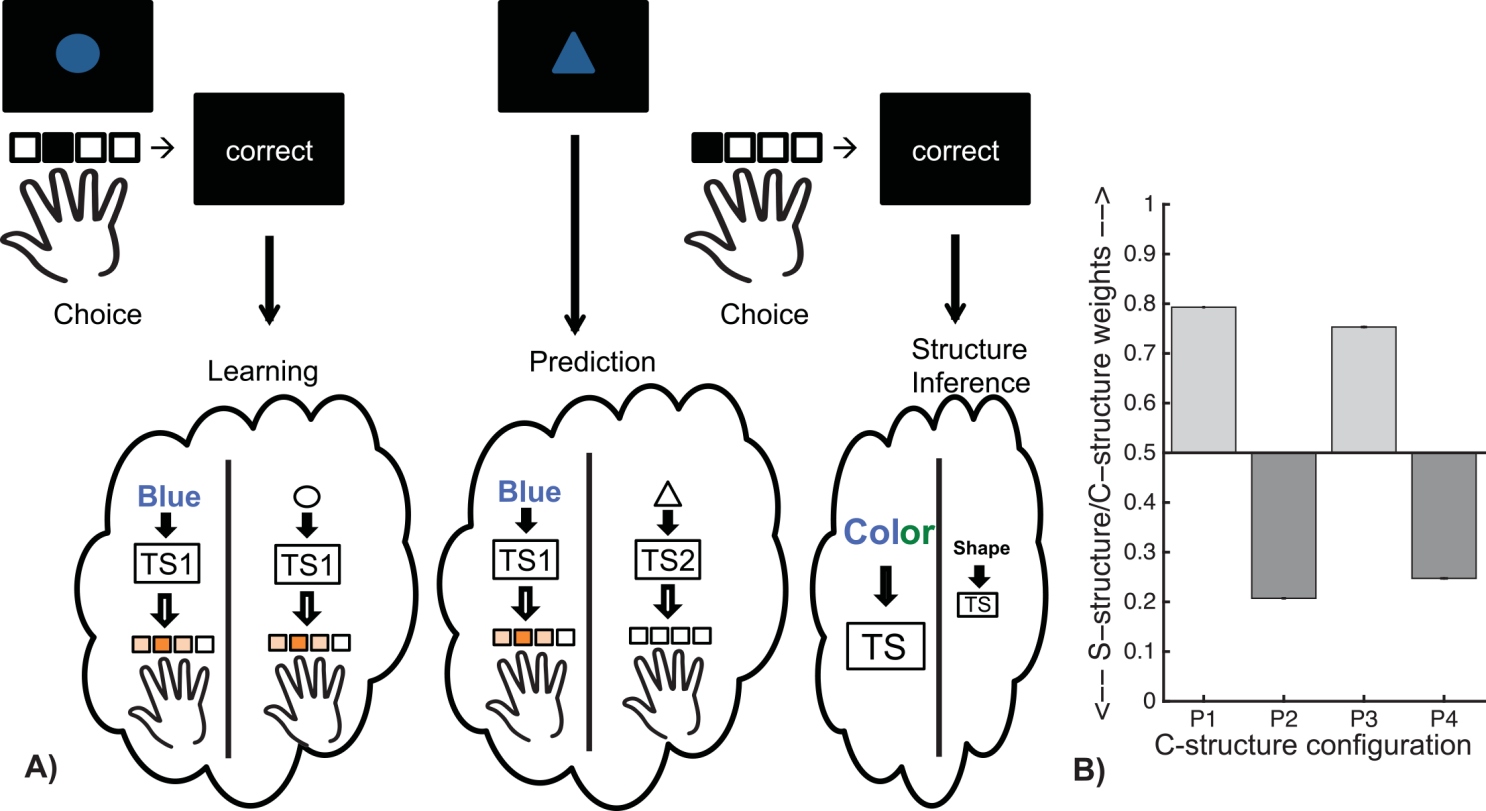
Computational model of motor clustering. **A)**: Example illustration of the prediction bias mechanism. Left: Given a blue circle, correct feedback induces learning of the corresponding action for the selected task-set, but also spreads across adjacent actions. This is illustrated for both possible structures, whereby either color or shape cues task-set. Middle: A blue triangle is presented. The color structure cues TS1 and induces outcome prediction biased to adjacent fingers; no such bias is predicted for the shape structure (triangle cues TS2). Right: following selection of left index finger, the observed correct outcome was better predicted by the color structure than the shape structure. Since the model selects structures according to how well they predicts outcomes, inference leads to a stronger belief in a color structure than shape structure. See methods and supplement for model equations and detailed description. **B).** Model simulation results (averaged over 1000 simulations–error-bars indicating standard error overlap with line width), average final inferred mixture weight w_C_ for C-structure (where color cues task-sets–see methods). Equal preference for either type of structure would be indicated by w_C_ = 0.5. X-axis indicates the color-structure configuration-pattern. When the C-structure leads to higher adjacency bonus patterns (P1,P3), the model is more likely to infer this structure as the correct to create, whereas when the C-structure leads to lower bias patterns (P2, P4), it is more likely to infer the other S-structure as valid.

Recall that by design, the experimental paradigm used here can be equally well described by either structure, with either color or shape acting as context cueing task-sets, and as such our previous model did not distinguish between them during learning [15]. Here we augmented the model to investigate the potential influence of motor clustering mechanisms (e.g., the overlap in encoding of motor actions by distributed neuronal populations [23,24]) that could explain the structure patterns observed empirically. Specifically, the model assumes that the hierarchical selection of a task-set constrains pre-activation of action representations available within this task-set, allowing the model to account for specific error patterns and hierarchical neural signals that are explained by such processing [15,22]. We now further assume that this activation spreads to adjacent motor actions, given their representational overlap (Fig 5A), and can bias outcome prediction within the selected task-set. Note that this proposed mechanism is *local*, in that it relies on the overlap of neuronal populations encoding adjacent fingers; but it requires that this overlap is used such that it can influence selection of the higher level task-set: implying a motor constraint on cognitive rule structure.

We simulated two versions of models including this bias: in one case, the bias was used only to influence action selection (comprising a pure motor bias during choice); in the other case the motor overlap was also used to predict outcomes of selected actions (see supplement for details). Simulation results of the former showed that it could not account for any structure preference: inferred weights were in average equal for both structures (w_C_ = w_S_ = 0.5). Indeed, a pure motor bias only affects which keys are pressed and does not lead to inference that one structure is better than the other, and hence predicts no asymmetry in switch costs, unlike the empirical data. In contrast, when we allowed the overlap in motor representations to also influence predictions (Fig 5 –right) this simple local mechanism allowed our model to infer that the best structure was that which afforded greater motor clustering (P1>P2, P3>P4). Indeed, it allowed those task-sets involving adjacent motor actions to better predict outcomes and hence the model identified that structure as better fitting the environment. Overall, these simulations confirm that scaffolding of higher-level abstract rule learning on low level motor representation can lead to biases in abstract structure representation, as observed empirically.

The above findings showed that subjects favor hierarchical rule structures that lead to heuristically simple motor patterns within each rule, in accordance with patterns of motor cortical representations. We next asked whether this representational bias is strong enough that it would influence subjects’ task representation even when learning was unnecessary and task structures are instructed.

**Fig 6:**
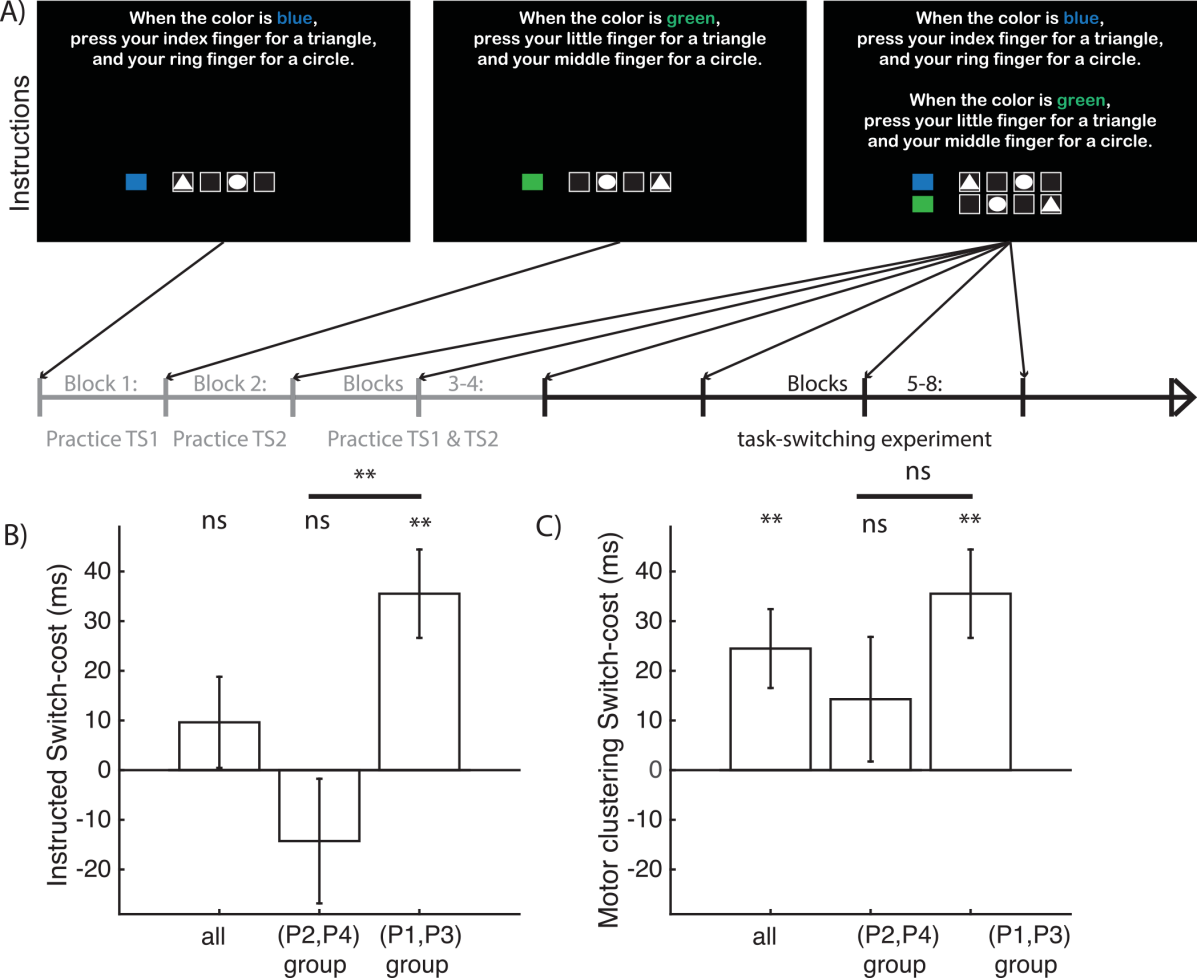
Instructed experiment. **A)** In this experiment, subjects did not have to learn the rules by trial and error, but were instructed the rules. The rule instructions and practice order were designed to favor a specific structure: subjects first practiced each task-set separately in two separate blocks, then they practiced them interleaved in two additional blocks. They were reminded of the instructions at the beginning of each block of the task-switching experiment. We show here instructions for configuration P4 as shown in Fig 2, with role of color and shape opposite compared to P4 in Fig 3. **B.** We computed instructed switch-cost as the reaction-time switch cost assuming the instructed structure representation of the task. Instructed switch-cost was not significant across all subjects. There was a significant difference between the group of subjects who were instructed rules that afforded more adjacency (P1,P3), who exhibited significant instructed switch-cost, and the group of subjects that were instructed rules that went against this adjacency bonus (P2,P4), who did not show an instructed switch-cost. **C.** We computed motor-clustering switch-cost as the reaction-time switch cost assuming that subjects structure the task according to the clustering bias, rather than the instructed structure. Motor-clustering switch-cost was significant across all subjects, and there was no significant difference between the two groups.

To this end, a separate set of subjects performed an instructed task-switching experiment in which they were told which dimension indicates the task-set and which constitutes the stimulus. Subjects were instructed the specific rules corresponding to one of each motor configuration pattern structure (groups for P1, P2, P3 and P4), and practiced those rules in a way that shaped the instructed structure (see Methods, Fig 6A). We were interested whether subjects that were instructed a rule that did not afford motor clustering (groups P2 and P4) would show signs of restructuring those rules to represent them instead within the corresponding motor clustering structure. If subjects followed the instructed representations, we expected that they should exhibit the standard reaction-time switch-cost corresponding to that instructed structure. In contrast, if the representational bias was strong enough, we expected that they would no longer show the classical instructed task-set switch cost because it would be offset (or even reversed) by the motor biases. Results showed that subjects in the P1/P3 group (allowing for motor clustering) had a significant instructed switch-cost (p = 0.002, t(11) = 3.99). In contrast, subjects in the P2/P4 group which did not afford motor clustering, did not show any instructed switch-cost (t(12) = -1.14), and this was significantly different from the other group (p = 0.004, t = -3.19, Fig 6B). Thus the classical task-switch cost was abolished when motor clustering favored the alternative structure. Indeed, over the whole group, instructed switch-cost was not significant (t(24) = 1.05). Instead, the whole group showed a motor clustering switch-cost (t(24) = 3.08, p = 0.005) with no significant difference between the instructed groups (Fig 6C t = -1.36, p = 0.19), lending support to the possibility that rather than performing the task with the instructed representations, subjects might have restructured their representation of the task according to the same motor biases observed in the learning experiment.

## Discussion

When learning to make choices, subjects search for structure and create representations that rely on hierarchical decision trees: at a higher level, they select abstract task-set rules based on contextual cues, which constrain how they select low-level motor actions in response to stimuli. When such a structure matches the statistical regularities of the world, subjects can discover it and leverage that structure to simplify the problem, speeding learning and facilitating transfer [15,19,32]. However, it is not always evident which structure is best. The results we presented here highlight a reversed hierarchical role in rule structure, where low-level features of the motor choices influence the nature of high-level task-set creation. We showed that a representational constraint of motor actions–adjacency overlap–influences the creation of rule structures: this bias manifests itself as a tendency to create rule structures that afford selection of adjacent motor actions for different stimuli within a same task-set. Indeed, we found across four independent data-sets that subjects were more likely to create a structure that afforded rules that primed adjacent motor actions. We showed in the last experiment that this bias was strong enough to offset classic reaction-time switch costs even in instructed task-switching experiments when the opposite structure afforded greater motor clustering.

These findings shed light on internal processes of how abstract rules are created and facilitate choice, even when such structure has no bearing on which motor responses are actually executed. Indeed, no matter what structure is used, the sequence of visual inputs and required motor actions is identical. The only difference elicited by distinct sorts of structure is observable in terms of how switching from one perceived ‘task’ to another elicits a cost, which in turn is related to the ability to transfer this task to novel contexts [15,22].

Our predominant hypothesis, indicating that executive functions are rooted in action selection, and that that implementations of decision making are scaffolded on motor circuits such that motor constraints affect cognitive processing, is inspired from a popular subfield of the *embodied cognition* literature [5–7,33] (though see [34] for other perspectives on what is required to be considered embodied cognition). Our neural network model of structure learning [15] hypothesized that task-set selection depended on hierarchical cortico-basal ganglia loops, with a prefrontal loop exerting control over a more posterior motor loop, but feedback from motor choices reciprocally reinforcing task-set selection in higher order loops. Here, we augmented an algorithmic version of this model to represent known features of motor cortex. Our model simulation showed that solely assuming facilitated action preparation could not account for preference in created rule-structures. Rather, our model accounts for the findings by assuming that overlap in motor patterns influences outcome predictions, such that structures involving motor clustering were better at predicting the observed outcomes within task-sets and hence inferred to be valid.

Another theory that could potentially account for the observed biases relates to spatial representation–rather than motor selection–biases. Indeed, it is possible that subjects represent the task-sets in a mental one-dimensional space (much as we represent it in the figures with a row of four squares), where a bias for adjacency in motor actions for a task-set would correspond to a spatial grouping bias (both actions on the left, in the middle, or on the right). Previous research in simpler decision-making tasks have shown that both motor and sensory biases could be important [28,29,35], particularly in conjunction, as proposed by Adam’s grouping model [28]. Disentangling both contributions here would require either systematically decorrelating the association between motor action selection and spatial action representation, or using neuroimaging to highlight the role motor cortex and overlap in motor representation play here. Both are beyond the scope of this study, but important targets for future research. However, it is important to note that such an interpretation could correspond to a visuo-motor process, involving for example eye movements, as suggested by research in other domains of high-level cognition (e.g. mental number line [36]), indicating that spatial biases do not rule out an embodied cognition interpretation.

We have focused here on motor adjacency as a low-level, sensory motor factor that influences abstract high level rule creation. Adjacency is well supported by potential mechanisms and neural data, and thus serves as a first intuitive example to establish this influence, inspired by the embodied cognition literature. However, we do not claim that it is the only factor; indeed other low level factors may also influence rule representation. As a preliminary example, we show in supplementary analysis that beyond similarity in motor space (summarized here by adjacency), similarity in the task-set space, as measured by a parallel or symmetric left-right association between stimuli and fingers, may also provide a bias on rule creation and can be captured by the same model (where symmetry influences prediction and inference). Furthermore, beyond such biases, statistics of the task itself can sometimes constrain which structures are more useful than others, e.g. when they facilitate better generalization (Collins & Frank submitted,[14]). But it is tempting to ask why, when the environment does not constrain abstract rule structure learning, low-level sensorimotor biases may do so. It is possible that this is a pure incidental byproduct of the architecture in which prefrontal-cortex learning and choice scaffolds on motor-based cortico-basal ganglia loops. However, it is also possible that this reflects the result of adaptive pressure: one might imagine that sets of rules used together in the same context tend to require more similar actions, and thus that it would be *a priori* useful to assume this kind of prior when learning novel structure. While this hypothesis resonates with embodied cognition literature, it remains speculative within the frame of this study.

Research in the field of cognitive control tends to think in abstract terms–of stimuli, choices, and values–disembodied from what they are–pictures or sounds, binary key-presses or joystick movements, points or food, assuming that this is processed independently upstream for inputs, and downstream for motor selection. The results presented here show that a very “low-level” feature–exactly with which finger presses choices were made–systematically influences how a very high-level, abstract representation is created, to the point of overwriting trained instructions. This highlights the need to pay careful attention to how sensory-motor factors may bias observed results, and supports the motor scaffolding theories of embodied cognition. It emphasizes how abstract representations that we build for high level-cognition and reasoning likely emerge from the constraints of interacting with connectivity to its input and output regions.

## Materials and Methods

### Task contingencies

In all tasks, for a given trial, subjects were presented with a single two-dimensional visual pattern on a black screen. There were two possible features on each dimension (e.g. Green and blue Color, triangle and circle Shapes), combining to form four distinct visual input patterns (Figs 1–3). Subjects could make one of four choices, but only one lead to *correct* feedback, while the others lead to *incorrect* feedback. The correct choice was different for each input pattern (Fig 2A). In learning experiments, input-pattern order presentation across trials was pseudo-randomized to ensure equal presentation, and equal frequency of first-order transitions. Subjects were instructed to use the four fingers of the right hand to select an action, except in the R-BL experiment (see below), where they used middle and index fingers of left and right hand to press four contiguous keys.

#### Clustering bonus

The clustering or adjacency bonus of each motor structure configuration is defined as follows. A task-set is grouped if actions for both stimuli require key presses from adjacent fingers. We assign an adjacency bonus of *a* = 1 for each task-set in the structure that is adjacent, otherwise *a* = 0. For example, for pattern P1 (see Fig 3), both task-sets are grouped, so that *a* = 2; in structure motor pattern P3, one task-set is grouped (cued by green), and the other is not, so that *a* = 1; in patterns P2-4, neither task-set is grouped, i.e., *a* = 0.

Note that, accounting for shape and color permutations, there are only 6 possible classes of patterns (S3 Fig). We analyze here only 4 types of patterns (P1-4, Fig 3), because predictions for the last type of pattern (P5-6) are confounded by other biases, such as symmetry biases. See supplement for more details.

#### Structure identification

We use reaction time switch-cost of each dimension as evidence in favor of either structure [15,22]. Specifically, we define the *motor clustering switch-cost* as the difference between the switch costs defined by the higher bonus structure and the one defined by the lower bonus structure, i.e. switch cost for P1 or P3 minus the switch-cost for P2 or P4. For the instructed experiment, in addition, we define instructed switch cost as the difference between the switch cost defined by the instructed structure and the one defined by the other potential structure (e.g. P1-P2 for a subject instructed P1, but P2-P1 for a subject instructed P2). All switch-costs were computed at asymptotic performance on correct trials, and we also controlled for sequential motor effects on reaction-times (see supplement).

#### Experiment specificities

All details can be found in supplement. We highlight here only the crucial points.

### Learning experiment

This experiment included twelve independent learning blocks, with non-overlapping sets of stimuli for each block. 6 of those blocks, which are analyzed here, corresponded to a different structure learning problem as defined in “Learning task rules” section, each with different non-overlapping stimuli; they included 80 trials each. 22 subjects performed this experiment, with 40 blocks of configuration P1/2, 59 of configuration P3/4 and 33 blocks of configuration P5/6 (not analyzed here). 18 subjects had at least one P1/2 block, and all subjects had at least one P3/4. For switch-cost difference analysis, results were first averaged across blocks within subjects per configuration, before group analysis. For binary structure assignment analysis, we included all 99 blocks of P1/2 and P3/4 configurations independently.

To replicate findings from this learning task, we analyzed three further data-sets with similar structure, but only a single block iteration of rule learning:

- R1 is the data set whose results were published in [15]: we showed that subjects created structure, and that reaction-time switch cost allowed us to identify which structure subjects created by showing that it predicted subsequent transfer. We test here whether motor patterns can predict which structure subjects build.
- R-EEG is the data set published in [22].
- R-BL is an unpublished data set, and differs from others only by use of 2 fingers of each hand, instead of 4 fingers of right hand, for key presses.

#### Instructed experiment

This experiment relied on the same contingencies described in the learning experiment, but did not necessitate learning. Instead, subjects were instructed the contingencies (Fig 6A) and practiced them in a way designed to shape their representation to a given task structure over blocks 1–4 (Fig 6A, see supplement). We analyze here blocks 5–8, which included 120 trials each, with a reminder of the instructions at the beginning of each phase. There were no repeat trials, and all transition types were equated. 37 subjects performed this task, with 6 subjects per group corresponding to each of the configuration types (except 7 subjects for configuration type P1). We thus analyze the data of 25 subjects.

#### Modeling overview

The model builds on that published in [15], as described in main text. To model effects of spatial motor patterns on structure learning, we introduce biases in the outcome prediction for the model’s representation of each potential structure. Specifically, we implement an adjacency bias by assuming that task-set selection may more broadly pre-activate action representations relevant to this task-set, independent of the specific current stimulus, including spreading to adjacent motor actions (see Fig 5, left). In brief, having selected an action according to a given structure for a specific stimulus biases neighboring actions to be predictive of corresponding outcomes even for stimuli that have not yet been encountered. The simulations shown here also include a symmetry bias (see supplement), though results for P1-4 are identical if we do not include this bias. Both biases are mixed evenly, then applied to the normal prediction of outcomes.

### Modeling details

Our model includes two separate “experts”, each of which considers one of the two possible structures (e.g. color as context expert, vs. shape as context expert, see Fig 5A). It then infers the most likely expert that best describes the data as a function of how well each expert predicts observed outcomes over time. Each expert rapidly learns to associate a given context to an abstract latent variable that represents the associated task-set and learns to predict outcomes (here reinforcement feedback), contingent on the current stimulus, chosen action and inferred task-set (rather than context). We first reiterate the model and then describe how we expand it to accommodate motor clustering effects.

Specifically, at each trial t, we label C_t_ and S_t_ the observed color and shape, with the chosen action a_t_, and r_t_ the obtained reward outcome. The color-structure expert learns to associate a color context C to an abstract, latent task-set variable Z, by keeping track of the probability of a given task-set given the color, P(Z|C). For a new context C_new_, the prior probability of a given task set is initialized following a Chinese Restaurant Process with concentration parameter *α* such that:

  $$P\left(Z_{new}|C_{new}\right)=\frac{\alpha }{\alpha +\sum P\left(Z_{i}\right)}$$
  $$P\left(Z_{i}|C_{new}\right)=\frac{P\left(Z_{i}\right)}{\alpha +\sum P\left(Z_{i}\right)}$$

Thus novel contexts are assumed to be most likely linked to existing task-sets that have been most popular across variable contexts, and with some possibility of creating a novel task-set.

Following observation of an outcome, the posterior probability P(Z|C) is updated according to Bayes rule, using learned likelihood *p*(*r*_*t*_|*S*_*t*_, *a*_*t*_, *Z*_*t*_). At each trial, Z_t_ is inferred using maximum a priori. The inferred Z_t_ constrains both the policy that is used for choice during the current trial, and learning of S-a-r contingencies:

- Choice policy *π*(*a*_*i*_|*Z*_*t*_, *S*_*t*_) is a noisy softmax on expected outcome *p*(*r*_*t*_ = 1|*S*_*t*_, *a*_*t*_, *Z*_*t*_)
- *p*(*r*_*t*_ = 1|*S*_*t*_, *a*_*i*_, *Z*_*t*_) is learned by tracking frequencies of each outcome, which is equivalent to a Bayesian updating of a beta distribution. (Note that we obtain similar results if we use a standard reinforcement learning delta rule instead.)

The shape-structure expert is identical to the color-structure expert, with the roles of color and shape reversed.

When both experts are included in the model, they both learn and select actions independently. However, the final action choice proceeds from the mixture of each expert’s policies: *π*(*a*) = *w*_*C*_(*t*)*π*_*C*_(*a*) + (1 − *w*_*C*_(*t*))*π*_*S*_(*a*), where the mixture weight *w*_*C*_(*t*) is the inferred reliability of each expert. It is initialized at *w*_*C*_(0) = 0.5, and updated via Bayes rule:

$$w_{C}\left(t+1\right)=\frac{w_{C}\left(t\right)\;p\left(r_{t}|S_{t},a_{t},Color\;Structure\right)}{w_{C}\left(t\right)\;p\left(r_{t}|S_{t},a_{t},Color\;Structure\right)+\left(1-w_{C}\left(t\right)\right)p\left(r_{t}|S_{t},a_{t},Shape\;Structure\right)}$$

In words, the expert that best predicts observed outcomes is assumed to be best for representing the current environment.

To model effects of spatial motor patterns on structure learning, we introduce biases in the outcome prediction of each expert. Specifically, we implement an adjacency bias by assuming that task-set selection may more broadly pre-activate action representations relevant to this task-set, independent of the specific current stimulus, including spreading to adjacent motor actions (see Fig 5, left). In brief, given that a task-set has been selected, the action-outcome contingencies learned are generalized to neighboring actions such that they become predictive of corresponding outcomes, even for stimuli that have not yet been encountered. This translates into a bias that modifies expected outcomes following:

$$bias\left(a|S_{t},Z_{t}\right)=\underset{i}{\sum }\pi (a_{i}|others\left(S_{t}\right),Z_{t})neighbor(a|a_{i})$$

where $neighbor\left(a|a_{i}\right)=\frac{1}{\#\;neighbors\left(a_{i}\right)}$ if a is a neighbor of a_i_, 0 otherwise; and *others*(*S*_*t*_) indicates the set of stimuli different from *S*_*t*_. This bias may be used in a mixture for policy selection and outcome prediction. We show in main text that a model using bias for only policy selection does not account for the empirical results, while a model using it for both does.

Biases are mixed to the normal prediction with mixture weight f_i_ (f_i_ = 0.1) in simulations. Other model parameters are:

- softmax parameter *β* = 30
- concentration parameter *α* = 2
- noisy action selection within task-set *ϵ* = 0.1

We simulate the model 1000 times and report asymptotic preference for either expert.

Although we assumed that motor overlap influences predictions, it is also possible that a similar effect results via imperfect credit assignment during learning. That is, when a given action is reinforced, the same overlap in motor representations could elicit partial reinforcement of adjacent motor representations. As such, the network would not only be more likely to select adjacent actions for the same task-set, but also would be better able to learn to predict accurate outcomes for a clustered task-set, leading to both better performance and greater reliability of an adjacent structure. Indeed, model simulations show that this mechanism produces similar results to the one exposed here. Our current findings cannot separate out these hypotheses, though we suspect that both could occur simultaneously (and indeed both theories rely on motor overlap).

## Supporting Information

S1 Text This supplementary information includes supplementary methods including detailed methods for each experiment. It also includes supplementary results, in particular validating the measure of adjusted reaction-time switch-cost.
(DOCX)

S1 Fig Corrected switch-cost predicts error types. Here 1and 2 indicate the two sensory input dimensions (e.g. color and shape); SwC indicates the reaction time switch-cost assuming that dimension as the high-level context dimension. NL indicates “neglecting low-level feature” error types, where the action selected was appropriate for this trial’s context, but not for the current stimulus. [15] showed that subjects are more likely to do NL than other types of errors. This result confirms that the corrected switch-cost difference is a reliable indicator of which structure subjects built.
(EPS)

S2 Fig Motor patterns and hierarchical structure. **A.** Correct action contingencies: visual input patterns are two-dimensional images, e.g. colored-shapes. **B** this can be represented hierarchically, with one of the features (here, e.g. color) serving as a context cueing a task-set (TS), and the other as a stimulus for which an action is selected given the TS. **C,D.** Depending on mapping of actions A1-A4 to individual fingers, different learning problems afford different motor patterns (in this case we show two problems both assuming the same hierarchical structure). **C.** In this configuration, motor patterns within both task-sets are “adjacent” (the correct key presses for stimuli are on adjacent fingers), leading to an *adjacency bonus* a = 1+1. The configuration is also symmetric (same left-right association for both task-sets), leading to a *symmetry bonus* of s = 1. **D.** Here, motor patterns required for both task-sets are *non-adjacent* (a = 0), and the configuration is asymmetric (opposite left-right associations across task-sets; s = 0).
(EPS)

S3 Fig Hierarchical structure representation affects motor patterns. For a single “abstract” learning problem (top left), different assignment of actions A1-A4 to actual finger presses F1-F4 (left), leads to different patterns of motor assignment across task-sets. A given assignment will lead to one of two possible configurations, depending on which structure is chosen (color on top or shape on top). **Bottom right.** Each motor configuration (P1-6) is depicted assuming subjects structure the task with task-sets selected according to color (P1-P3-P5 on left) or shape (P2-P4-P6 on right), with the context that cues each task-set shown (eg–blue for TS1 of P1, triangle for TS1 of P4). For each pattern, the adjacency bonus *a* (indicating whether a task-set’s actions are adjacent) and symmetry bonus *s* (indicating whether both task-sets dictate the same left-right arrangement of both stimuli) are indicated, and their sum is natural bias score n, in red. Note in particular that neighboring configurations (P1 and P2, P3 and P4, P5 and P6) correspond to the same colored-shape action contingencies, grouped into different hierarchical structure representations, and that P1 and P3 have a higher natural motor bonus than their counterpart. Role of color and shape is counterbalanced to ensure that the color-as context structure does not always correspond to clustering configurations P1/P3/P5, as is shown here.
(EPS)

S4 Fig Subjects favor creation of structure corresponding to motor clustering configuration. **Left column. Motor clustering bonus, in terms of switch-costs differences**, for each experiment and configuration type. Markers indicate individual subjects, bars are group average, errorbars correspond to standard error. **Right columns**. Proportion of learning blocks identified as represented with easy (dark bars) or hard (light) structure. **Top line**. Experiments 1–3 show a strong effect of easy vs. hard, indicating that subjects tend to create the heuristically more intuitive structure. There is no effect of experiment (see main text). **Middle line** shows that this is true for both configurations (no effect of configuration type). **Bottom line** shows that experiment 4 replicates previous findings, with more data points per subject and better correction of switch-cost for potential sequential motor effects.
(EPS)

S5 Fig Computational model of motor clustering. **A)**: illustration of the prediction bias mechanism. The model selects a structure according to how well each structure predicts outcomes. After a correct choice (e.g. index finger for a blue triangle), the prediction is defined by the learned values for the current task-set and stimulus (triangle for P2, blue for P5). If an adjacency bias is included, the prediction for the current stimulus (smaller blue triangle) is biased evenly for adjacent actions within the same task-set (here cued by blue for P2, triangle for P5), applied to other stimuli (here circle for P2, green for P5). If a symmetry bias is included, it is biased towards action positions in proportion to how likely they are to lead to symmetric configurations, based on knowledge for the same stimulus in the other task-set. We highlight here the potential symmetric configurations for this situation, showing that the prior for the true position of the current image (blue triangle) is 1/3 for P2, 2/3 for P5. Overall, the outcome predictions are a mixture of the biases and normal predictions (mixture parameter ɛ). Overall, the observed outcome is slightly better predicted by P2 than P5, which over time leads to the inference that P2 is the better structure.
(EPS)

S6 Fig Task-switching experiment. Examples of instruction screen including both task-sets, for different configurations (see main text Fig 6). Note that the specific role of green vs. blue, triangle vs. circle, and even color vs. shape were randomized across subjects, so this is only one type of possible instructions per configuration.
(EPS)
