# Supplementary material for: Motor Demands Constrain Cognitive Rule Structures
Source: PLoS Comput Biol. 2016 Mar 11;12(3):e1004785. doi: 10.1371/journal.pcbi.1004785 (PMC4788431; doi:10.1371/journal.pcbi.1004785)
Supplement: S1 Text — It also includes supplementary results, in particular validating the measure of adjusted reaction-time switch-cost. (DOCX) [file pcbi.1004785.s001.docx]

Motor demands constrain cognitive rule structures:

Supplementary information

## Anne GE Collins, Michael J Frank

# Supplementary methods

## Experimental design

Main learning experiment:

This experiment included twelve independent learning blocks, with non-overlapping sets of stimuli for each block. 6 of those blocks (called 2-d blocks) had the structure as described in the main text, and included 80 trials each. In the other 6 blocks (called 1-d blocks), subjects learned problems with 4 non-overlapping stimuli (e.g. four different colors). We do not analyze those blocks here, as they are not relevant for the question related to task-sets, in which features overlap for different task sets. We used all 2-d blocks’ pooled data to estimate finger-to-finger transition costs on reaction times for each subject, thus correcting for potential sequential motor effects before computing reaction-time switch costs. These costs were evaluated over the second half of each block, during which performance was asymptotically high. 22 subjects performed this experiment, with 33 blocks of configuration P1/4, 40 of configuration P2/5, and 59 of configuration P3/6. 18 subjects had at least one P2/5 block, and all subjects had at least one P3/6. For the switch-cost difference analysis, results were first averaged across blocks within subjects per configuration, before aggregate group analysis. For the binary structure assignment analysis, we included all 99 blocks independently.

Results from the replication learning experiments (statistics reported in the main text) are plotted in figure S4. Specific details for methods relating to these experiments follow.

Replication Learning Experiment #1 (R1):

The data from experiment 1 was previously published in (1), but without analysis of motor pattern effects. This re-analysis focuses on the initial learning phase, which corresponds to one learning block of the main learning experiment, and to the design described in the main text. The learning phase included a minimum of 40, and a maximum of 120 trials, up to a performance criterion, followed by a 40 trial asymptotic phase, over which reaction time switch-cost was computed. Stimulus presentation was pseudo-randomized to equate the presentation of each of the four visual inputs, and to balance switch and stay on each dimension.

Due to counterbalancing of other task-factors across subjects for that study (such as role of color/shape as context/stimulus, and input-pattern trial sequences), the association between actions and motor patterns was kept similar across subgroups of subjects, leading to an imbalanced distribution across configuration types. Out of 32 subjects included, 26 had configuration 1/2, and 6 configuration P5/6. Thus, the current analysis of this task includes 26 subjects.

Replication Learning Experiment # 2 (R-BL):

Experiment design was similar to previous experiment, with the difference that index and middle fingers from both hands were used for choice. 67 subjects performed the task, with 21 subjects for configuration P5/6, 22 subjects for configuration P1/2, and 24 for configuration P3/4. We thus analyze 46 subjects here.

Replication Learning Experiment # 3 (R-EEG):

Results from experiment 3 were previously published in (2), where full methods can be found. Design differed from experiment 1 by the jittered timing of stimulus presentation required for EEG analysis. We only include the first iteration of the learning task in this analysis. Out of 32 included subjects, 10 had configurations P5/6, 13 configuration P1/2, and 9 configuration P3/4. The present analysis of this task thus includes 22 subjects.

Instructed experiment:

This experiment relied on the same contingencies described in the learning phase of experiment #1, but did not necessitate learning. Instead, subjects were instructed the contingencies (figure 6) and practiced them in a way designed to shape their representation to a given task structure. Specifically, subjects first read instructions for a single context (eg. the color blue), and were visually and verbally instructed on what key to press for each stimulus in this rule. They then practiced this rule for 20 trials. They then repeated the same procedure for a second rule. Blocks 3 and 4 reminded subjects of both instructions, then proceeded to let them practice the contingencies for 40 trials each. In block 3, a given rule applied for 10 consecutive trials before switching. In block 4, the rules applied for 5 consecutive trials. Blocks 5-8 included 120 trials each, with a reminder of the instructions at the beginning of each phase. There were no repeat trials, and all transition types were equated. For reaction-time switch cost computation, we included only correct and preceding correct trials, and excluded the first 3 trials of each block. 37 subjects performed this task, with 6 subjects per group corresponding to each of the configuration types (except 7 subjects for configuration type P2). We thus analyze the data from 25 subjects, with 12 instructed the “easy” configurations P1/3, and 13 instructed the difficult configurations P2/3.

## Reaction time Switch Costs

To infer which type of structure is used, we rely on the reaction time switch-cost, where greater switch costs following changes in color than shape are indicative of use of color to cue the task-set, and vice versa. We have previously validated this measure in that it was predictive of subjects’ ability to transfer the structure to novel contexts that involve the same structure, and was also predictive of subjects attention to the high level context as decoded from neural signals (1,2). Nevertheless (as noted in the main text) the decision of which dimension was considered high level context was arbitrary. Here we assess whether this switch cost asymmetry can be predicted by motor clustering bonus. To do so, we calculate switch cost for P2 or P3 minus the switch-cost for P5 or P6. Switch-costs were computed over correct trials of an asymptotic performance task for learning experiments 1-4, and over all correct trials of blocks 5-8 in instructed experiment 5 (excluding the first 2 trials of each block).

It is important to note that other factors such as sequential motor transitions could influence RTs, which might confound our measures. For example, after using the index finger to press a key at trial t subject might be faster to press the key associated with the middle finger than the ring finger at trial t+1. We therefore took care to ensure that such motor transition costs would not confound our findings, given that different structures might have different transitions.

We indeed identified that while the fastest transitions were action repeats, action switches to adjacent fingers were faster than those to more distant fingers (“jumps”), which could confound switch-cost comparisons between patterns. Specifically for a middle previous action (middle or ring finger), RT slowing to the furthest finger (little or index finger, respectively) was significantly higher than to the other middle, adjacent finger (ring or middle finger respectively; p = 0.001, t=3.77). For a trial where the previous action was an “extremum” finger press, jumps two or 3 fingers away were significantly slower than switches to the adjacent finger (3: p=.067, t = 1.93; 4: p = 0.006, t=3.03), and they didn’t differ from each other (t=1.03).

Thus, to ensure the switch-cost difference measure is not biased due to low-level motor sequence effects, we correct for the effect of jumps before computing switch-cost. In the replication learning experiments and the instructed experiment, we do so by comparing trials that afford identical jump conditions

In the main learning experiment, since subjects performed many learning blocks with different configurations, we could separate more directly the role of motor transitions from the role of task and stimulus switches to reaction-times. Specifically, we computed the average reaction times for all potential action transitions (mRT(ij) is the average reaction time in trial t+1 when action for trial t is I, and action for trial t+1 is j), and then corrected reaction times by the appropriate mRT(ij) for all trials, based on what the correct actions were for the successive trials. Thus, reaction time switch cost computation were corrected for specific key-press transitions.

## Overall natural bonus: adjacency and symmetry

In the main text, we focused exclusively on adjacency, as a potential sensorimotor influence on structure learning. However, other factors may also be important. Here we highlight a notion of parallelism across task-sets, or symmetry, which we propose could also involve an analogous type of representational overlap at the task-set level, as opposed to the above-described motor cortical overlap between adjacent finger representations. We combine adjacency and symmetry in a single metric, the “overall natural bonus”, that indicates which structure configuration is expected to be more natural, including both adjacency and symmetry biases.

As noted in main text, there are only 6 possible patterns of structure configurations (figure S3). We focused on four of them in the main text (P1 trough 4), because they made similar predictions whether we included symmetry bonus or not. For completeness, we include here the additional configurations P5 and P6.

**Overall natural bonus.** The overall natural bonus of each motor structure configuration is defined as follows (see figure S2):

- *adjacency bonus*: as defined in main text. A task-set is grouped if actions for both stimuli require key presses from adjacent fingers. We assign an adjacency bonus of *a*=1 for each task-set in the structure that is adjacent, otherwise *a*=0. For example, for patterns P1 and 5, both task-sets are grouped, so that *a*=2; in structure motor patterns P3 and 6, one task-set is grouped (cued by green and circle respectively), and the other is not, so that *a*=1; in patterns P2 and 4, neither task-set is grouped, i.e., *a*=0.
- *Symmetry bonus*: a configuration is considered symmetrical if both task-sets correspond to the same left-right relative positioning of the correct actions across stimuli. We assign a bonus *s* = 1 for symmetrical patterns (P1, P2, P6) and a bonus of *s*=0 otherwise.
- The *overall natural bonus n* of a motor pattern configuration induced by a structure is defined as the sum: *n* = *a*+*s*, and indicated in red in figures 2, 3. Note that other combinations of *a* and *s* could be chosen. For example, one might argue that n= .5**a*+*s* might be a better score, as it would equate the scale of both bonuses. We do not commit particularly to the chosen form here, but think it is the most a priori logical one, as one could expect adjacency to matter more, the more task-sets were involved. Note that this only matters for configurations P5-6, which is why we chose to exclude them from main text analysis.

Since P5 and P6 lead to equally natural biases (n=2), we restrict our analysis to learning problems where the finger assignment was such that the two possible configurations were either P1/P2 or P3/P4, for all 5 data-sets. For P5/6, experimental results are consistent with equal bias in that we observe no preference for either in any experiment. However, as this is a null result, it should not be taken as direct evidence and is not included in main text.

**Computational model: symmetry bias.**

The symmetry bias is implemented similarly to the adjacency bias (see main text methods), but with respect to the other task-set and same stimulus (rather than same task-set and other stimulus):

P_bias_(r_t_,a|S_t_,Z_t_) = Σ_i_ pi(a_i_| S_t_, other(Z_t_))*sym(a|a_i_)

where sym(a|a_i_) indicates the likelihood that this action a can lead to a symmetric pattern with a_i_. Simple enumeration (see figure S5 for symmetry bias intuition) shows that

- sym(a|a_i_ = 1) = [0 2/3 1/3 0] (and reversed for a_i_ = 4).
- sym(a|a_i_ = 2) = [2/3 0 0 1/3] (and reversed for a_i_ = 3).

In model simulations including both biases, adjacency and symmetry biases are mixed evenly, then mixed to the normal prediction with mixture weight f_i_ (f_i_ = 0.1). Main text simulations (fig 5) include both biases, though results are qualitatively identical if only adjacency bias is included. The same results are plotted in figure S5 bottom, including configurations P5-6.

# Supplementary results

## Corrected RT-Switch cost validation

As noted above, we had previously shown that RT-switch-costs were a good measure of which structure was used by a subject, since they predicted future transfer based on that structure (1) – thus, we verified that we saw the same relationship with this RT-switch-cost now corrected for jumps. Indeed, we found that the corrected switch cost predicted subjects’ subsequent ability to transfer a learned task-set to a new context (r=0.47, p = 0.006; note that the correlation is numerically stronger than we previously obtained with uncorrected switch cost), confirming that it was an appropriate indicator of the nature of the structure subjects built.

We also found in the main learning experiment that corrected switch-cost predicted other behavioral measures indicative of structure (1), such as error type repartition (figure S1; Spearman rho=0.44, p <10^-6^), further confirming that subjects were building structure in this experiment and that the corrected measure was a good indicator of which structure they built

## Main experiment – other indicator of structure building.

See figure S1 below.

## Learning experiment – Three replication data sets.

See figure S4 below, and main text for statistics.

Bibliography

1. Collins AGE, Frank MJ. Cognitive control over learning: Creating, clustering, and generalizing task-set structure. Psychol Rev. 2013;120(1):190–229.

2. Collins a. GE, Cavanagh JF, Frank MJ. Human EEG Uncovers Latent Generalizable Rule Structure during Learning. J Neurosci. 2014 Mar 26;34(13):4677–85.
